# Finite element stress analysis of the bearing component and bone resected surfaces for total ankle replacement with different implant material combinations

**DOI:** 10.1186/s12891-021-04982-3

**Published:** 2022-01-19

**Authors:** Jian Yu, Dahang Zhao, Wen-Ming Chen, Pengfei Chu, Shuo Wang, Chao Zhang, Jiazhang Huang, Xu Wang, Xin Ma

**Affiliations:** 1 Department of Orthopedics, Huashan Hospital, Fudan University, 12 Wulumuqi Zhong Lu, Shanghai, 200040 China; 2 Department of Orthopedics, Ruijin Hospital, Shanghai Jiaotong University, 197 Ruijin Er Lu, Shanghai, 200020 China; 3 Academy for Engineering and Technology, Fudan University, 220 Handan Lu, Shanghai, China

**Keywords:** Computational modeling, Finite element method, Implant design, Total ankle arthroplasty, Total ankle replacement, Implant material selection

## Abstract

**Background:**

A proper combination of implant materials for Total Ankle Replacement (TAR) may reduce stress at the bearing component and the resected surfaces of the tibia and talus, thus avoiding implant failure of the bearing component or aseptic loosening at the bone-implant interface.

**Methods:**

A comprehensive finite element foot model implanted with the INBONE II implant system was created and the loading at the second peak of ground reaction force was simulated. Twelve material combinations including four materials for tibial and talar components (Ceramic, CoCrMo, Ti6Al4V, CFR-PEEK) and three materials for bearing components (CFR-PEEK, PEEK, and UHMWPE) were analyzed. Von Mises stress at the top and articular surfaces of the bearing component and the resected surfaces of the tibia and talus were recorded.

**Results:**

The stress at both the top and articular surfaces of the bearing component could be greatly reduced with more compliant bearing materials (44.76 to 72.77% difference of peak stress value), and to a lesser extent with more compliant materials for the tibial and talar components (0.94 to 28.09% difference of peak stress value). Peak stresses at both the tibial and talar bone-implant interface could be reduced more strongly by using tibial and talar component materials with smaller material stiffness (7.31 to 66.95% difference of peak stress value) compared with bearing materials with smaller material stiffness (1.11 to 24.77% difference of peak stress value).

**Conclusions:**

Implant components with smaller material stiffness provided a stress reduction at the bearing component and resected surfaces of the tibia and talus. The selection of CFR-PEEK as the material of tibial and talar components and UHMWPE as the material of the bearing component seemed to be a promising material combination for TAR implants. Wear testing and long-term failure analysis of TAR implants with these materials should be included in future studies.

**Supplementary Information:**

The online version contains supplementary material available at 10.1186/s12891-021-04982-3.

## Introduction

Total ankle replacement (TAR) has become an effective procedure for end-stage ankle arthritis which replaces damaged ankle with artificial implants to restore ankle function [1]. The early and midterm clinical results of new-generation ankle implants appear promising [2, 3]. However, the implant survivor rate of TAR is not comparable with that of total knee arthroplasty (TKA) or total hip arthroplasty (THA) [4–7]. The common post-operative complication of TAR includes implant failure of the bearing component and aseptic loosening at the bone-implant interface [8–10]. The mechanism behind it is still unclear, and biomechanical factors may play an important role in the cause of these complications [11–13]. Therefore, it is needed to further understand the biomechanics of the TAR to help the design of new total ankle implants with improved clinical performance.

The materials selection for implant components are critical to implant design and proper material selection may avoid local excessive bone stress [14–16]. However, no consensus of the optimum material combination for total ankle implant has been reached [17]. Most existing total ankle implants used ultra-high molecular weight polyethylene (UHMWPE) as bearing material and metal (cobalt–chromium–molybdenum alloy (Co-Cr-Mo) or Titanium alloy (Ti6Al4V)) as the material of tibial or talar components. Only one TAR system chose to use ceramic as tibial and talar component material (TNK ankle, Kyocera, Kyoto, Japan) [18]. Polyether-ether-ketone (PEEK) and carbon-fiber-reinforced Polyether-ether-ketone (CFR-PEEK) have been used in joint replacement as a potential alternative to both the metal and bearing components [19, 20]. However, studies about the biomechanics of the TAR implant with different materials are lacking.

Due to the difficulties in measuring the stress on the implant and bones, finite element (FE) simulation had been widely used for the pre-clinical biomechanical evaluation of orthopedic implants [21, 22] and a few FE studies tested the effect of implant material for TAR [12, 23–30]. Kerschhofer et al. [29] simulated contact stress and wear rate of PEEK and its composites on Wright State University (WSU) TAR devices. Through a tibial bone-implant construct of the STAR ankle system, Mondal et al. [14] investigated the relationship between implant materials and several biomechanical parameters (tibial strain, micromotion, and wear depth) in a FE hindfoot model. However, the above-mentioned studies only considered partial models of the foot. The load and boundary conditions were not realistic. Considering the complexity of ankle anatomy, a comprehensive foot model accounting for ligaments, cartilage, muscular force, and 28 bones of the foot and ankle may better recreate the biomechanics of the ankle in silico. Ozen et al. [31] first developed a FE model of the whole foot and ankle implanted with the Bueshel-Pappas implant system at a balance standing position to simulate the stress at each bone. More recently, Wang et al. [12, 30] use a comprehensive FE foot model to compare the difference of the plantar pressure, joint contact pressure, the peak stress value among an intact foot, a foot with STAR ankle system, and a foot after ankle arthrodesis.

**Fig. 1:**
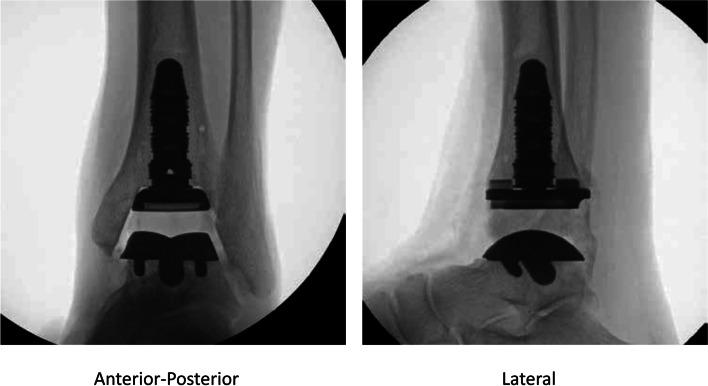
X-ray images of the INBONE II implant system. Intra-operative anterior-posterior and lateral fluoroscopic images of the ankle with INBONE II implant system. (Image courtesy of Wright Medical Technology, Inc., Memphis, TN, US)

In this study, a comprehensive FE foot model implanted with an INBONE II implant system (Wright Medical Technology, Inc., Memphis, TN, US) was constructed to investigate the biomechanical impact of different implant material combinations on the stress distribution at the bearing component and the bone-implant interface. It was hypothesized that the material of components with smaller material stiffness may reduce the peak stress at their nearby surfaces. We chose to place this implant system into the intact FE foot model (see Fig. 1) since it is the only ankle implant system currently used in China. It has a medullar segmented stem, assembled with a tibial tray, and a symmetric fixed bearing component with full conformity to the talus component [32]. To the best of the authors’ knowledge, no studies have previously investigated the biomechanical performance of this ankle system.

## Method

### FE modeling of the foot and implant constructs

The current FE model was modified from the model used in previous studies [33, 34]. All bones were connected by 134 major ligaments and a plantar fascia. The ligaments were represented by spring elements with a ‘no compression’ option. The 3D geometries of the plantar fascia and Achilles tendon were constructed. The other five muscle tendons, namely tibialis posterior (TIBP), flexor hallucis longus (FHL), flexor digitorum longus (FDL), peroneus brevis (PB), and peroneus longus (PL), were also incorporated into the model using bar elements, at their corresponding anatomical attachment sites.

The plantar soft tissue was modeled by an incompressible Ogden hyperelastic material [33]. The strain energy function U of the first-order Ogden model was defined by:

$$\mathrm{U}=\frac{2\mu }{\alpha^2}\left({\lambda}_1^{\alpha }+{\lambda}_2^{\alpha }+{\lambda}_3^{\alpha }-3\right)$$

The material constants *μ* and *α* were determined by a previous in-vitro study [35] and equaled 3.75 × 10^−2^ MPa, and 5.5, respectively. Isotropic linear elastic material properties were assigned to the bones [36], ligaments [37], cartilages [38], plantar fascia [39], Achilles tendon [33, 40] and flexor tendons [41]. The details of the material parameters used in the foot model are given in Table 1. All 3-D structural components of the model were meshed by linear tetrahedral elements (C3D4) with a maximum edge length of 2.3 mm determined in previous studies [33].

**Table 1:** Material property and element type of the foot model

	Elastic modulus (MPa)	Poisson ratio	Cross-sectional area (mm2)	Element type
Bone	7300	0.3	–	4-node tetrahedron
Cartilage	1.01	0.4	–	4-node tetrahedron
Ligament	260	0.4	18.4	Spring
Plantar Fascia	350	0.4	–	4-node tetrahedron
Achilles tendon	816	0.3	–	4-node tetrahedron
Flexor tendon	450	0.3	12.5	Connector
Plantar soft tissue	1st Ogden incompressive hyperelastic model *μ* =0.0375 MPa, *α* =5.5			4-node tetrahedron
Ground	Rigid			8-node hexahedron

### TAR implant modeling and implantation

To match the shape of the current foot model, the INBONE II implant system with a size two long tibial component, a size two polyethylene insert with 6 mm thickness, and a size two talar component was used. The geometry of this implant system was reconstructed by manual measurement of the dimensions of the implant components retrieved from patients who performed TAR revision surgeries. The standard materials of the tibial component, bearing, and talar component of INBONE II implant system were Ti6Al4V, UHMWPE, and CrCoMo, respectively. The tibia and talus were resected with the protection of the medial malleolus, and this ankle system was implanted following its operative guideline [42] from the manufacturer. The implantation procedure was guided and checked by two senior foot and ankle surgeons (X.M. and X.W.). The mesh size of linear tetrahedral elements for the implant was determined by the mesh convergence test (Supplementary Table S1), which was 1 mm mesh size at the implant articular surface and 1.5 mm at the rest of the implant surface.

**Fig. 2:**
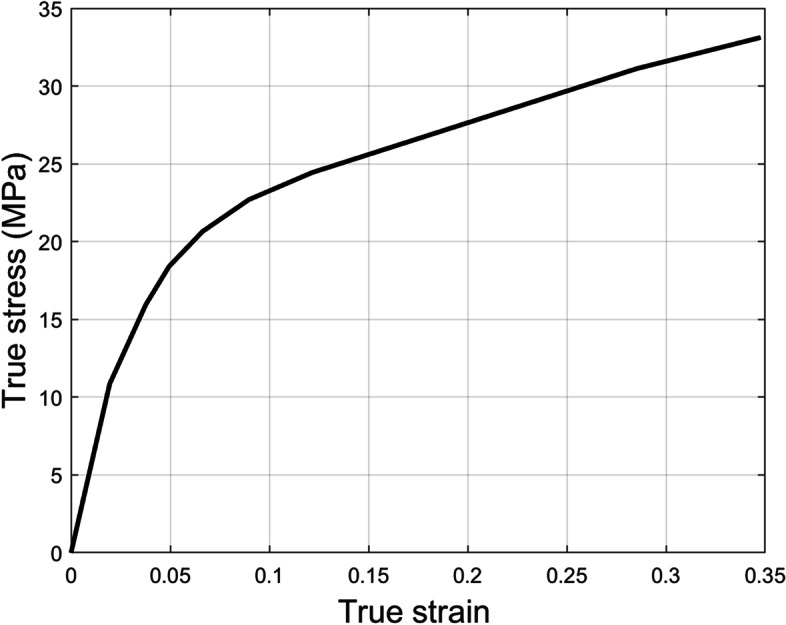
The stress-strain curve of the UHMWPE material [43]

**Fig. 3:**
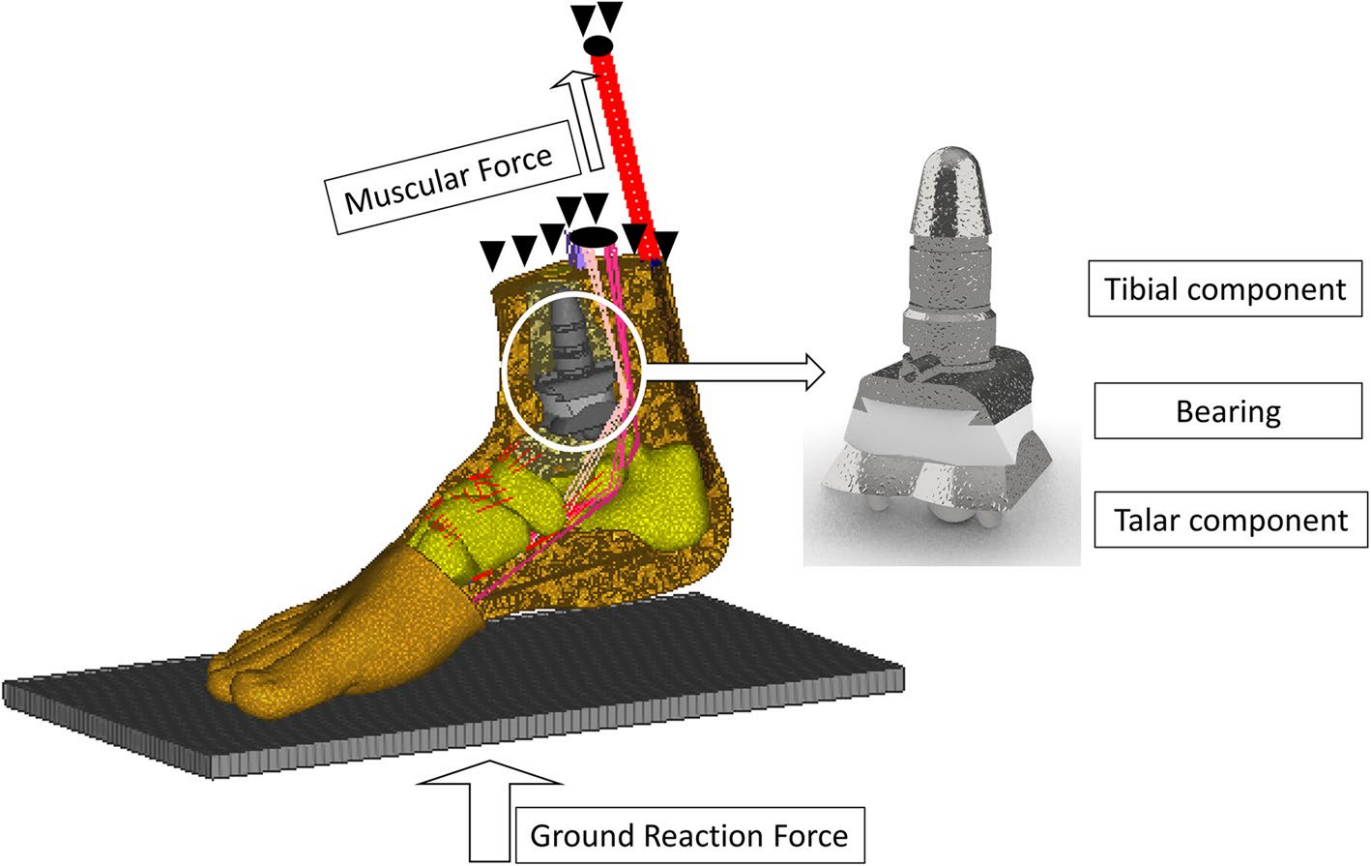
An illustration of the finite element foot model implanted with INBONE II implant system (Tibial component size: size 2 long. Bearing size: size 2 polyethylene insert with 6 mm thickness. Talar component size: size 2)

In this study, four types of materials were used to model tibial and talar components (Ceramic, Co-Cr-Mo, Ti6Al4V, and CFR-PEEK) while three types of materials were used to model the bearing component (CFR-PEEK, PEEK, and UHMWPE). Therefore, the effect of a total of 12 combinations of implant materials was analyzed. To better mimic the biomechanical behavior of UHMWPE material, it was modeled as an isotropic elastic-plastic material [43] with elastic modulus of 556.92 MPa, yield stress of 10.86 MPa, and Poisson’s ratio of 0.46. The stress-strain curve of the UHMWPE is presented in Fig. 2. The rest of the implant materials were modeled as isotropic elastic materials [29, 44], and their mechanical properties are listed in Table 2**.** And the complete model is illustrated in Fig. 3.

**Table 2:** Material property used for implant material modelling

Implant Material	Elastic modulus (MPa)	Poisson’s ratio (ν)
Ceramic	350,000	0.26
Co-Cr-Mo	210,000	0.3
Ti6Al4V	114,000	0.342
CFR-PEEK	18,000	0.4
PEEK	4100	0.36
UHMWPE	556.92 MPa Stress-strain relationship [43]	0.46

### Loading and boundary condition

A finite-sliding surface-to-surface contact behavior was defined to all contact pairs. Normal (“hard” contact with default constraint enforcement method (linear penalty method)) and tangential (penalty or frictionless friction formulation) contact definitions were assigned for interaction properties. ABAQUS automated contact algorithm was used to define contact stiffness and penetration tolerance. A coefficient of friction (COF) of 0.5 was used for the frictional contact interaction between the plantar surface and the ground [33]. The articulation between the bearing and talus was defined as frictionless [31]. The interaction between bone and implant was defined as a tie condition to simulate fully ingrown bones. The tibial tray and bearing component were tied since it is the fix-bearing design.

We analyzed the biomechanical characteristics of the TAR implant at a time when the magnitude of ankle force reached a peak, namely the second peak of the ground reaction forces (GRF) in the normal gait cycle. A rigid plate under the foot was used to model the ground support. The ground was constrained to only allow movement in a vertical direction. The loading condition has previously been established in our foot FE model [33, 34]. In brief, a targeted maximum vertical GRF (623.1 N for the subject with a bodyweight of 60 kg) was generated solely by contracting plantar flexors corresponding to the push-off phase in gait. The obtained convergent solution of the muscle forces that maintain the second metatarsal shaft orientation was 1620 N of GS complex, 267 N of the TIBP muscle, 130 N of the FHL muscle, 81 N of the FDL muscle, 91 N of the PB muscle, and 193 N of the PL muscle. Muscle forces of major plantar flexors were applied via the tendons attached. To only investigate the impact of implant material, we assumed all models shared the same boundary and loading conditions.

### Data Analysis

All FE models were solved in ABAQUS (Simulia Corp., Providence, RI, USA). To validate the model by comparison with previously reported data, the plantar pressure and von Mises stress at the implant articular surface of an additional model with the standard materials of INBONE II implant system were simulated. The ankle force was computed by the summation of the magnitude of contact normal force (CNORMF in ABAQUS) of all nodes on the articular surface of the bearing component. For each implant material combination, the von Mises stress at the bearing component and the resected surfaces of the tibia and talus were evaluated and compared.

## Result

**Fig. 4:**
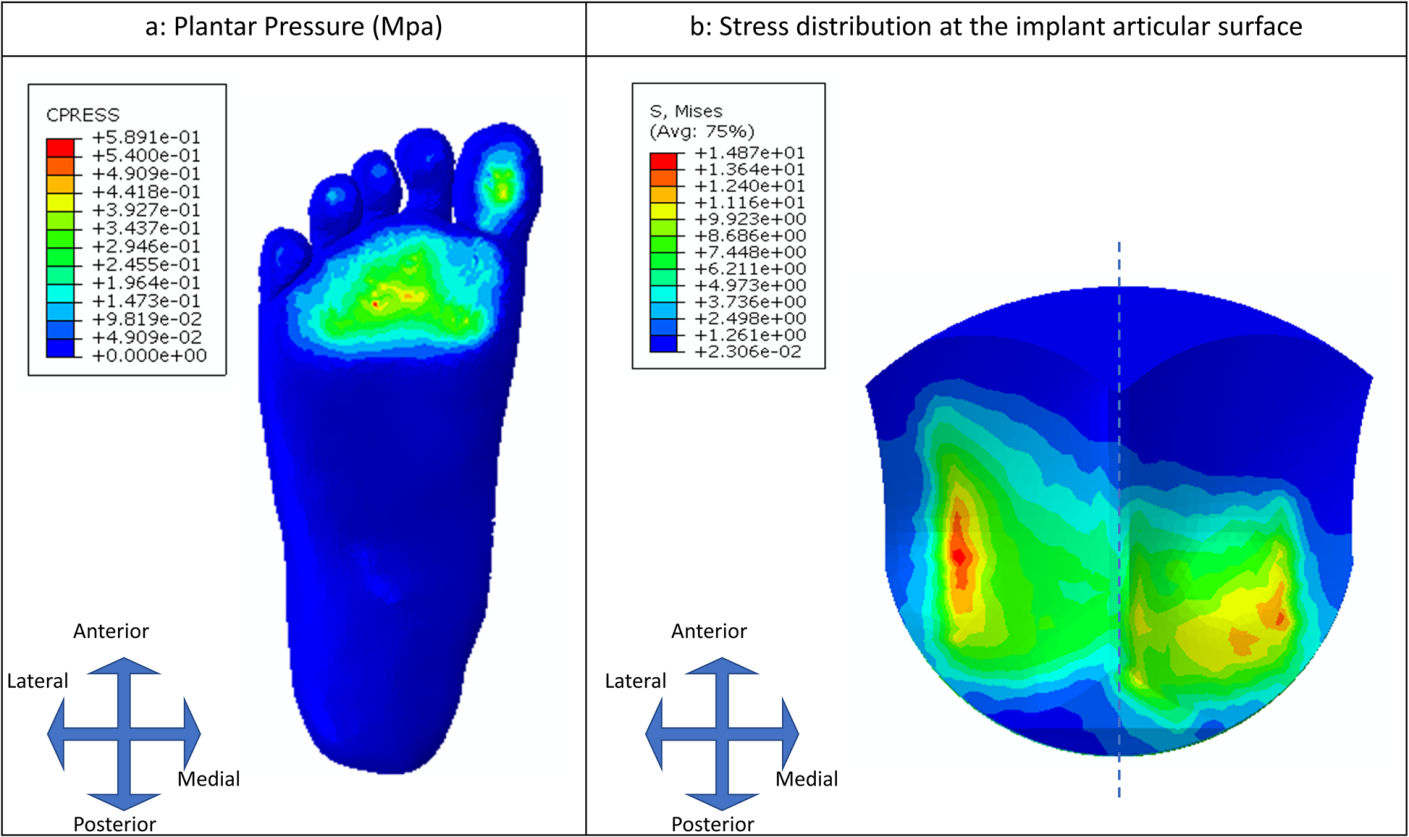
The plantar pressure of the foot (**a**) and von Mises stress distribution at the articular surface of the bearing component (**b**) (Implant material: Ti6Al4V as tibial component material, UHMWPE as bearing material, and CrCoMo as talar component material)

The plantar pressure of the foot and von Mises stress of the implant articular surface are plotted in Fig. 4. It can be seen that at the second peak of the GRF, plantar pressure concentrates at the forefoot and the plantar surface of the first toe **(**Fig. 4a**)**. The peak plantar pressure of the INBONE II implanted foot was 0.589 MPa, which was similar to that of the foot replaced with STAR total ankle system (0.605 MPa) [12]. The ankle joint typically experiences a higher joint force than the knee and hip joint during the gait, which can reach as much as 5–7 times of body weight [37, 45]. The total ankle force of the current model was 3463.2 N, which was 5.89 times body weight (BW) and was consistent with the literature. The total lateral force at the articular surface (1814.9 N) was predicted to be higher than the medial force (1648.3 N) (Fig. 4b), which was in good agreement with the simulation results of a musculoskeletal modeling study [46].

### Comparison of von Mises stress distribution at the resected surface of the tibia for different material combinations

**Fig. 5:**
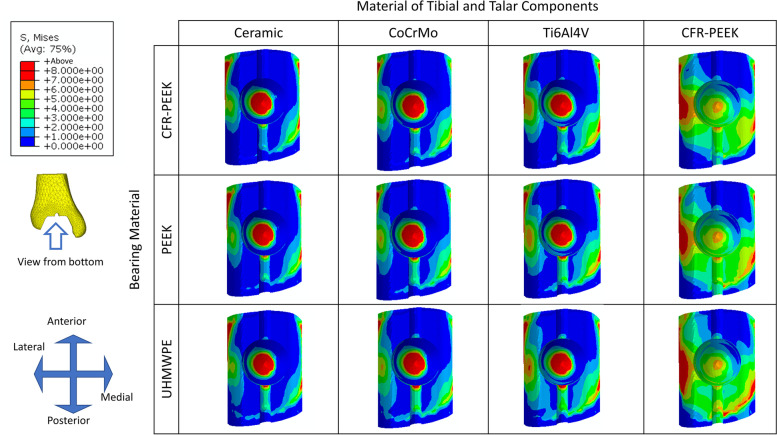
The von Mises stress distribution at the resected surface of the tibia for different implant material combinations

The predicted results of the stress distribution and peak stress value at the resected surface of the tibia for different implant material combinations showed that as the material stiffness of tibial and talar components decreased, the peak stress was largely reduced (66.70 to 82.12%) and stress distributed more uniformly (Fig. 5 and Table 3). The stress was insensitive to bearing material stiffnesses (the maximum difference of peak stress value ranged from 0.92 to 6.11%).

**Table 3:** Peak von Mises stress at the resected surface of the tibia for different implant material combinations

Peak von Mises stress (MPa)		The material of Tibial and Talar components				Maximum difference
		Ceramic	CrCoMo	Ti6Al4V	CFR-PEEK	
Bearing Material	CFR-PEEK	64.21	50.92	29.91	11.4	66.70%
	PEEK	64.23	50.86	29.84	12.12	80.59%
	UHMWPE	63.64	50.35	29.48	11.38	82.12%
Maximum difference		0.92%	1.12%	1.44%	6.11%	

### Comparison of von Mises stress at the top surface of the bearing component for different material combinations

**Fig. 6:**
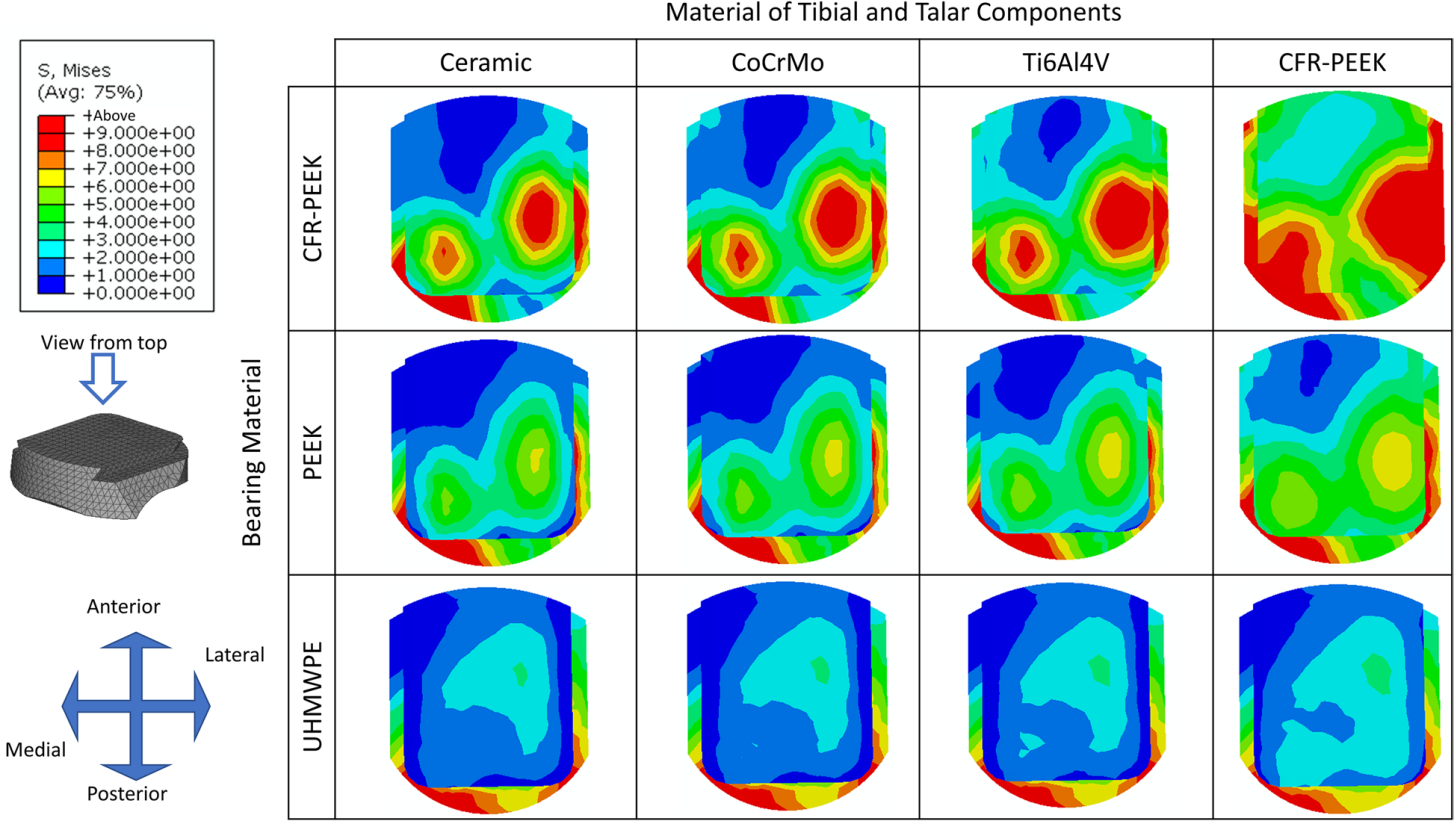
The von Mises stress distribution at the top surface of the bearing component for different implant material combinations

The predicted results of the stress distribution and peak stress value at the top surface of the bearing component for different implant material combinations showed a general trend that peak stress at the top surface of the bearing component decreased with the stiffness of both the implant and bearing materials, while the stress distributed more evenly (Fig. 6 and Table 4**).** When the material stiffness of tibial and talar components decreased, the peak stress was slightly decreased (The maximum difference of peak stress value ranged from 6.76 to 24.77%). However, as the material stiffness of bearing components decreased, the peak stress was largely reduced (44.76 to 66.95%).

**Table 4:** Peak von Mises stress at the top surface of the bearing component for different implant material combinations

Peak von Mises stress (MPa)		The material of Tibial and Talar components				Maximum difference
		Ceramic	CrCoMo	Ti6Al4V	CFR-PEEK	
Bearing Material	CFR-PEEK	23.58	22.57	21.32	17.74	24.77%
	PEEK	16.79	16.36	15.78	13.99	16.68%
	UHMWPE	10.51	10.46	10.32	9.80	6.76%
Maximum difference		55.42%	66.95%	51.59%	44.76%	

### Comparison of von Mises stress at the articular surface of the bearing component for different material combinations

**Fig. 7:**
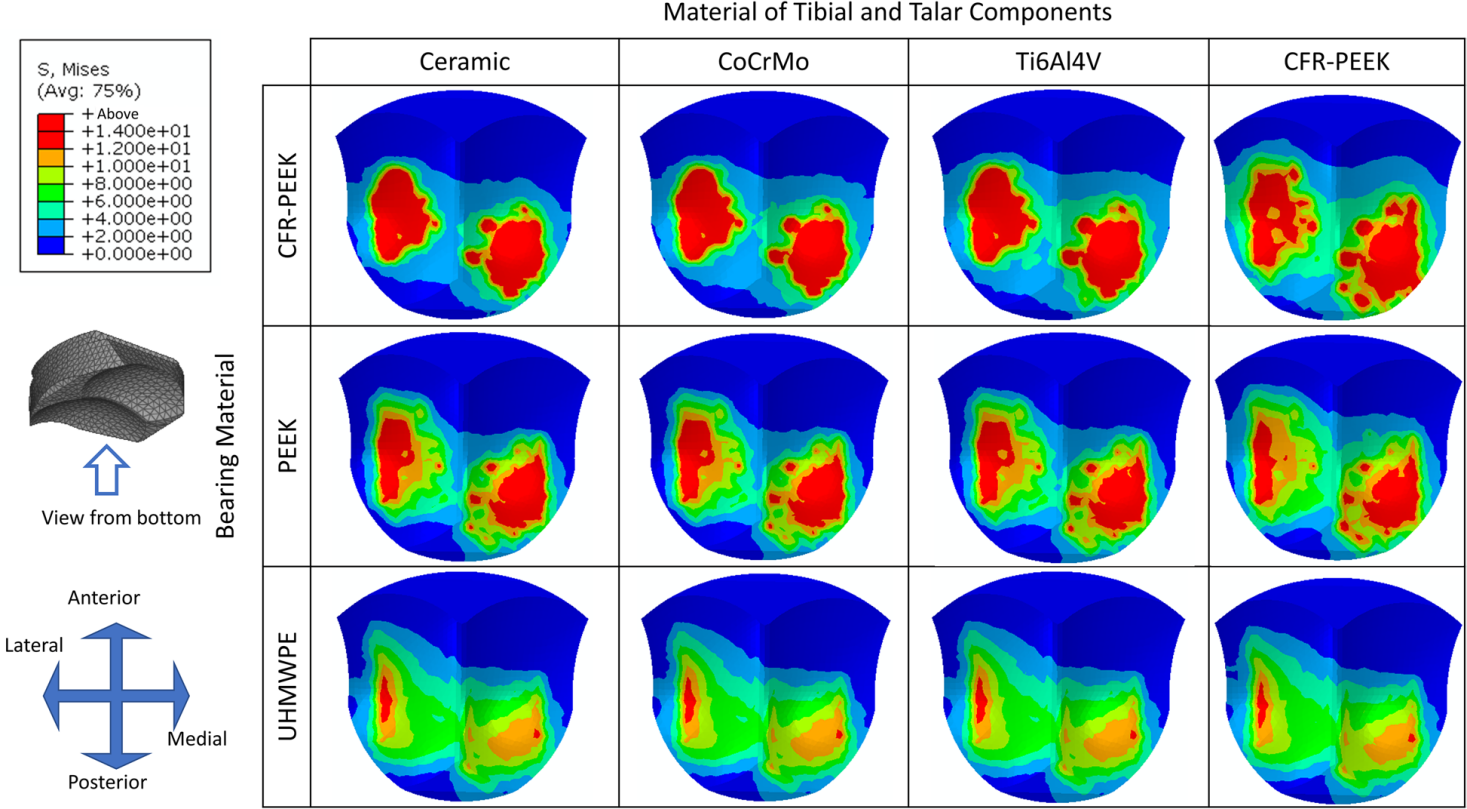
The von Mises stress distribution at the articular surface of the bearing component for different material combinations

Similar to the top surface, peak stress at the top surface of the bearing component decreased with the stiffness of both the implant and bearing materials (Fig. 7 and Table 5**).** The maximum stress reduction of changing the material of the bearing component from CFR-PEEK to UHMWPE was 59.79 to 72.77%, while that of changing the material of tibial and talar components was 0.94 to 28.09%.

**Table 5:** Peak von Mises stress at the articular surface of the bearing component for different material combinations

Peak von Mises stress (MPa)		The material of Tibial and Talar components				Maximum difference
		Ceramic	CrCoMo	Ti6Al4V	CFR-PEEK	
Bearing Material	CFR-PEEK	51.26	49.39	47.06	36.86	28.09%
	PEEK	29.83	29.41	28.84	27.26	8.62%
	UHMWPE	14.96	14.92	14.86	14.82	0.94%
Maximum difference		72.77%	69.79%	68.43%	59.79%	

### Comparison of von Mises stress distribution at the resected surface of the talus for different material combinations

**Fig. 8:**
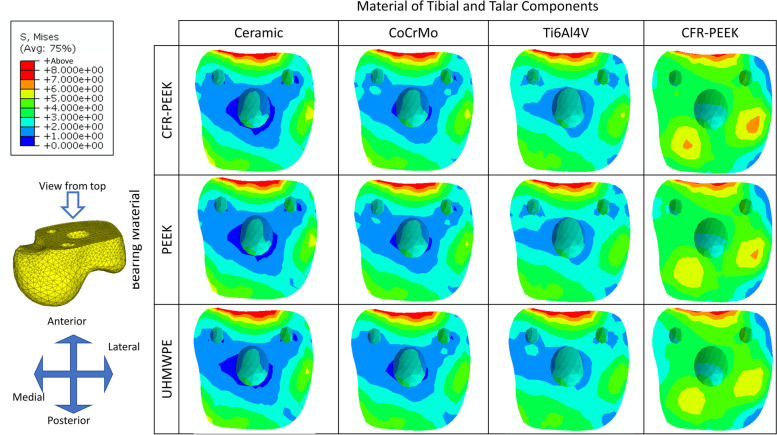
The von Mises stress distribution at the resected surface of the talus for different implant material combinations

The predicted results of the stress distribution (Table 6) and peak stress value **(**Fig. 8**)** at the resected surface of the talus for different implant material combinations showed that as the stiffness of tibial and talar components decreased, the peak stress was slightly reduced. The maximum stress reduction of changing from ceramic to CFR-PEEK ranged from 7.31 to 8.81%. The peak stress was insensitive to bearing stiffnesses (the maximum difference of peak stress value was all less than 5%).

**Table 6:** Peak von Mises stress at the resected surface of the talus for different implant material combinations

Peak von Mises stress (MPa)		The material of Tibial and Talar components				Maximum difference
		Ceramic	CrCoMo	Ti6Al4V	CFR-PEEK	
Bearing Material	CFR-PEEK	9.068	9.070	9.028	8.312	7.31%
	PEEK	9.068	9.051	9.000	8.373	7.66%
	UHMWPE	9.415	9.271	9.101	8.586	8.81%
Maximum difference		3.69%	2.37%	1.11%	3.19%

## Discussion

In this study, a comprehensive FE foot model implanted with the detailed geometry of the INBONE II ankle implant was constructed and loading at the second peak of the ground reaction force was simulated. The finite element model with this implant system has not been tested in previous studies. Twelve implant material combinations were applied to the implant components. The von Mises stress at the top and articular surface of the bearing component and the resected surface of the tibia and talus were analyzed and compared.

Our results revealed that the stress at both the top surface and articular surface of the bearing component can be greatly reduced with more compliant bearing materials (44.76 to 66.95% and 59.79 to 72.77% difference of peak von Mises stress value, respectively), and to a lesser extent with more compliant materials for the tibial and talar components (0.94 to 28.09% and 6.76 to 24.77% difference of peak von Mises stress value, respectively).

Bearing material with UHMWPE showed the lowest peak von Mises stress at the articular surface, which may be one reason why UHMWPE is commonly used as the bearing material. The combination of CFR-PEEK as the material of tibial and talar components and UHMWPE as the bearing material presented the lowest stress of 14.82 MPa at the articular surface, which closely matched the peak stress of the INBONE II implant system with the standard material property (14.87 MPa). However, the peak von Mises stress at the articular surface of bearing component at the second peak of GRF were all above the yield limit provided in the literature [43], which could be one of the reasons that UHMWPE material presents a high wear rate than PEEK and its composites in one simulation study [29]. Although improved manufacturing techniques enabled new UHMWPE material with higher yield stress [47, 48]. The bearing component with these materials should be further evaluated in wear analyses.

In addition, material change generally made an impact only on the adjacent structure. Peak stresses at the both tibial and talar bone-implant interface could be reduced more strongly by using tibial and talar component materials with smaller material stiffness (44.76 to 66.95% and 7.31 to 8.81% difference of peak von Mises stress value, respectively) compared with the bearing material with smaller material stiffness (6.76 to 24.77% and 1.11 to 3.69% difference of peak von Mises stress value, respectively). The tibial and talar component with a smaller material stiffness can smooth the stress change across the resected surface of the tibia and talus, thus CFR-PEEK seems to be a good biomechanically alternative to metal components. However, future studies should perform more comprehensive evaluations of the PEEK composite before its application to clinical practice.

The metal-on-metal implant configuration is not considered in this study, since no such arrangement has been used in existing ankle replacement implant systems, and concerns have been raised about its use in hip and knee arthroplasty by researchers [49] and the FDA of United States [50].

One major limitation of the study is that several assumptions were made for simplification, such as applying the fully ingrown conditions at the bone-implant interface without considering implant loosening at the initial stage, no separation of cortical and trabecular bone, neglecting the viscoelastic material property of all structures and the bone remodeling process, and simplified contact analysis, which may affect the accuracy of stress prediction, especially at the implant articular surface. What’s more, the current foot model was developed from CT images of one healthy volunteer and replaced by one implant model. Future studies should consider using the radiological data of more patients with the surgical implication of TAR. Although the current model was validated for plantar pressure and metatarsal bone strains, further validation of the model in cadaveric or in-vivo studies is still needed. Finally, stress analysis is the first step of the biomechanic study for material selection of TAR implants. Von Mises stresses alone can not fully evaluate the biomechanic performance of TAR implants. A more detailed contact, wear and long-term failure analysis should be included in future studies.

## Conclusion

Implant with smaller material stiffness could provide a stress reduction at both the bearing surfaces and the resected surfaces of the tibia and talus. UHMWPE as the material of the bearing component at the top surface presented the lowest peak value at the top and articular surfaces of the bearing component, while CFR-PEEK as the material of the tibial and talar component exhibited the lowest peak stress value at the resected surface of the tibia and talus. Thus, the selection of CFR-PEEK as the material of tibial and talar components and UHMWPE as the bearing material seemed to be a promising material combination for TAR implant, from the biomechanical perspective. Such results provided insight into the biomechanics of the TAR procedure and material selection of implant components. The FE foot model with the INBONE II implant system can be utilized for further biomechanical investigations of TAR and implant optimization, eventually benefiting patients who need TAR in clinical practice.

## Supplementary Information

**Additional file 1: Supplementary Table S1.** Sensitivity analysis of the effect of mesh size on the peak stress value (bold mesh size was the mesh size used in this study).

## Data Availability

The datasets used and analyzed during the current study are available from the corresponding author on reasonable request.
