# Supplementary material for: Finite element stress analysis of the bearing component and bone resected surfaces for total ankle replacement with different implant material combinations
Source: BMC Musculoskelet Disord. 2022 Jan 19;23:70. doi: 10.1186/s12891-021-04982-3 (PMC8772082; doi:10.1186/s12891-021-04982-3)
Supplement: Supplementary file 1 — Additional file 1: Supplementary Table S1. Sensitivity analysis of the effect of mesh size on the peak stress value (bold mesh size was the mesh size used in this study). [file 12891_2021_4982_MOESM1_ESM.docx]

**Supplementary Information**

**Supplementary Table S1.** Sensitivity analysis of the effect of mesh size on the peak stress value (bold mesh size was the mesh size used in this study)

| Implant Material Combination (Ti6Al4V+UHMWPE+CrCoMo) | | | | | | | |
| --- | --- | --- | --- | --- | --- | --- | --- |
| Mesh size at the articular surface of the bearing component (mm) | 1.5 | 1.5 | 1 | **1** | 1 | 0.8 | 0.8 |
| Mesh size at other surface of the implant (mm) | 1.8 | 1.5 | 1.8 | **1.5** | 1 | 1 | 0.8 |
| Total element number | 546180 | 546224 | 547933 | 548599 | 571717 | 573144 | 577704 |
| (1) Peak stress at the resected surface of the tibia (MPa) | 29.53 | 29.54 | 29.11 | 29.48 | 29.47 | 29.49 | 29.49 |
| (1) relative difference with the next mesh size | 0.03% | 1.46% | 1.26% | 0.03% | 0.06% | 0% | / |
| (2) Peak stress at the top surface of the bearing component (MPa) | 10.18 | 10.96 | 10.34 | 10.91 | 11.47 | 11.92 | 11.92 |
| (2) relative difference with the next mesh size | 7.12% | 5.66% | 5.22% | 4.88% | 3.78% | 0% | / |
| (3) Peak stress at the implant articular surface (MPa) | 13.00 | 13.06 | 14.13 | 14.87 | 14.49 | 14.12 | 13.68 |
| (3) relative difference with the next mesh size | 0.07% | 7.57% | 4.98% | 2.56% | 2.55% | 3.12% | / |
| (4) Peak stress at the resected surface of the talus (MPa) | 9.254 | 9.238 | 9.36 | 9.27 | 9.278 | 9.26 | 9.26 |
| (4) relative difference with the next mesh size | 0.17% | 1.30% | 0.96% | 0.09% | 0.19% | 0% | / |
